# *Gabrb2* knock-out mice exhibit double-directed PMDD-like symptoms: GABAAR subunits, neurotransmitter metabolism disruption, and allopregnanolone binding

**DOI:** 10.18632/aging.204351

**Published:** 2022-10-24

**Authors:** Mingzhou Gao, Hao Zhang, Ya Sun, Zhan Gao, Chunyan Sun, Fengqin Wei, Dongmei Gao

**Affiliations:** 1 Innovation Research Institute of Traditional Chinese Medicine, Shandong University of Traditional Chinese Medicine, Jinan, Shandong Province, China; 2 Experimental Centre, Shandong University of Traditional Chinese Medicine, Jinan, Shandong Province, China; 3 College of Traditional Chinese Medicine, Shandong University of Traditional Chinese Medicine, Jinan, Shandong Province, China

**Keywords:** premenstrual dysphoric disorder, allopregnanolone, GABAAR, neurotransmitter, subunits

## Abstract

Background: Premenstrual dysphoric disorder (PMDD) is a severe mood disorder with pathological changes rooted in *GABRB2* copy number variation. Here, we aimed to elucidate the gene dose effect and allopregnanolone binding mechanism of Gabrb2 on possible PMDD-like and comorbid phenotypes in knockout mice.

Methods: PMDD-like behaviors of *Gabrb2*-knockout mice were measured through various tests. Western Blot and ELISA were used to detect changes in the *GABAAR* subunits and related neurotransmitter changes in mice respectively for the internal mechanism. The response of mice to allopregnanolone (ALLO) was examined through an exogenous ALLO injection, then validated by the patch-clamp technique to elaborate the potential mechanism of ALLO-mediated *GABAAR*.

Results: *Gabrb2*-knockout mice displayed changes in anxiety-like and depression-like emotions opposite to PMDD symptoms, changes in social, learning, and memory capacities similar to PMDD symptoms, and pain threshold changes opposite to PMDD symptoms. *GABAAR δ* subunit expression in the brains of the *Gabrb2*-knockout mice was significantly higher than that of Wild-type mice (P<0.05). *Gabrb2*-knockout mice demonstrated neurotransmitter metabolism disturbance of GABA, Glu, acetylcholine, DA, norepinephrine, and epinephrine. Moreover, *Gabrb2*-knockout mice did not display the expected phenotypic effect after ALLO injection. Relative to WT mice, the knockout of the β2 subunit gene enhanced the agonistic effect of ALLO on GABAA receptors in cortical neuronal cells.

Conclusions: *GABAAR β 2* regulates PMDD-like behaviors. The ALLO binding site may not be located on *β two* subunits, abnormal δ and ε subunit expression in the mouse brain and the disturbance of neurotransmitters may result in ALLO sensitivity.

## INTRODUCTION

Premenstrual dysphoric disorder (PMDD) is a severe form of premenstrual syndrome (PMS), targeting a substantial portion of the female population [1, 2]. Clinically, PMDD is characterized by significant emotional, physical, and behavioral distress during the late luteal phase that resolves after the onset of menses [3]. Premenstrual disorders are likely to start at a younger age, particularly in adolescence [4, 5]. Many risk factors contribute to the development of PMDD. For instance, traumatic events, pre-existing anxiety disorders [6] and depressive disorder history, or a family history of PMS represent high-risk groups for suicidality [7–9].

Recent literature hypothesized that PMDD pathophysiology is caused by an impaired GABA_A_ receptors (GABA_A_Rs) response to dynamic ALLO fluctuations across the menstrual cycle [10], which primarily occur in the brain [11]. Moreover, neuroimaging has revealed greater cerebellar grey matter volume and metabolism in patients with PMDD, together with altered serotonergic and GABAergic neurotransmission [12], and other brain areas are also involved [12–14]. The role of GABA_A_Rs in the brain, particularly the subunit function, has attracted research interest [15, 16]. Among subunits of GABA_A_R, the copy-number-gains of GABRB2, genes encoding GABA_A_Rs β2 subunit have been enriched in both SCZ and PMDD patients with significant odds ratios (OR) [17]. And rat models of PMS showed abnormal expression of GABA_A_Rs β2 subunit in the hippocampus [18]. Also, GABRB2 is associated with other neuropsychiatric disorders, including bipolar disorder, epilepsy, autism spectrum disorder, Alzheimer's disease, frontotemporal dementia, substance dependence, depression, and internet gaming disorder [19–21]. Since GABRB2 has an important role in the central nervous system and contributes to human diseases, a better understanding of its function may speed up the search for novel therapeutic strategies.

The present study aims to compare the *Gabrb2*-knockout mice with wild-type mice regarding their associated PMDD-like phenotypes. Also, we evaluate the potential mechanisms of *Gabrb2* targeting ALLO.

## MATERIALS AND METHODS

### Animals

**Figure 1:**
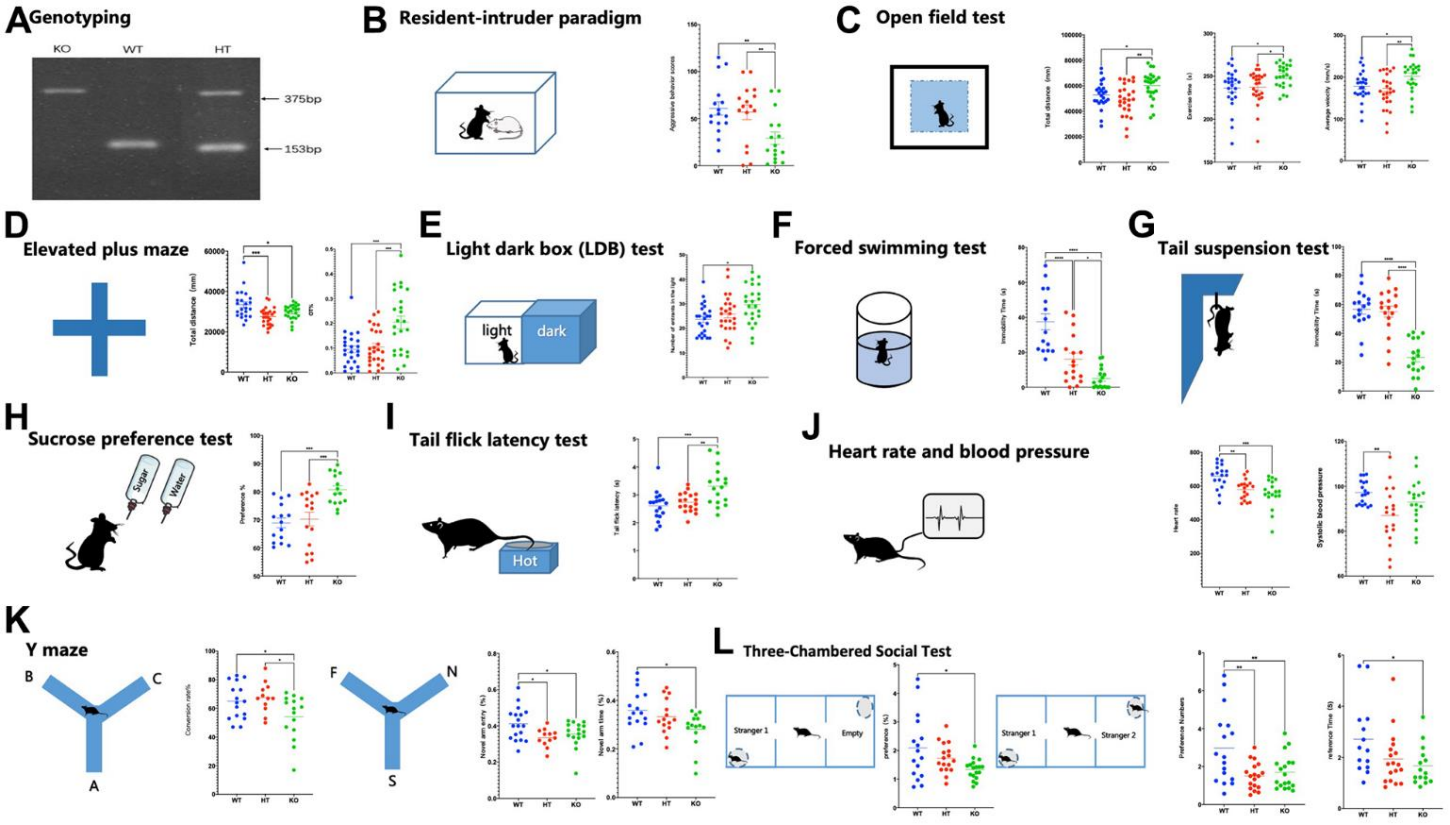
**Genotyping, affective symptoms, and PMDD-like behaviors of mice.** (**A**) Mouse genotyping using primers specific for the Gabrb2 and Neo genes. (**B**) Resident-intruder paradigm showing aggressive behavior scores of mice (WT male = 16, HT male= 16, KO male=16). (**C**) Open field test showing total distance, total distance in the central area, and total time in the central area (WT male =24, HT male=25, KO male=24). (**D**) Elevated plus maze showing percentile entries and time into open arms (WT male =24, HT male=25, KO male=24). (**E**) Light dark box (LDB) test showing entries and time into lightbox (WT male = 18, HT male= 18, KO male= 18). (**F**) Forced swimming test showing immobility time of mice suspended in the water (WT male =15, HT male=17, KO male=17). (**G**) Tail suspension test showing immobility time of mice suspended by the tail to a horizontal bar (WT male =17, HT male=17, KO male=17). (**H**) Sucrose preference test showing (WT male =15, HT male=16, KO male=15). (**I**) Tail flick latency test showing (WT male =18, HT male= 17, KO male=16). (**J**) Heart rate and blood pressure (WT male =18, HT male=18, KO male=18). (**K**) Y maze contains two parts: spontaneous alternation and novelty arm, showing percentile time spent in, or entries into, the novel arm was monitored to measure spatial-working memory (WT male =18, HT male=12, KO male= 17). (**L**) Three-Chambered Social Test contains two parts: social affiliation and social novelty, showing a preference for the container holding a stranger mouse relative to an empty container; and preference for social novelty: preference for the container holding a Stranger-2 mouse relative to the container holding Familiar-1 mouse (WT male =18, HT male=18, KO male= 18). Statistical analysis was performed using one-way ANOVA with Newman–Keuls post-hoc test. Average y values ± SEM in the different plots are represented by horizontal bars. *p < 0.05, **p < 0.01, ***p < 0.001.

*Gabrb2* heterozygous mutant (HT) transgenic mice on C57BL/6-129/SvEv hybrid background was provided by Professor Hong Xue’s team from the Department of Life Sciences at Hong Kong University of Science and Technology. The experiments used three genotypes based on the propagation of HT, wild-type (WT), and *Gabrb2* knockout (KO) mice by genotyping according to the previous protocol (see Figure 1A) [22]. The mice were housed in the laboratory at a temperature of 23 ± 3° C, a humidity of 60 ± 5% RH, 12 h/12 h light/dark cycle (lights on at 7 am and lights-off at 7 pm), with free access to water and food. The genotypes of each mouse were identified before experiments. The experimental procedures were approved by the ethics committee of Shandong University of Traditional Chinese Medicine (Permit Number: SDUTCM20190904013).

### Behavioral tests

Male mice (8-10 weeks) were selected for behavioral tests according to previous research [22, 23]. In the experiment, the “open field test-elevated plus maze-light and dark box” were used for multi-aspect evaluation of the anxiety-like emotional performance. The forced swimming-tail suspension-sucrose preference tests were adopted for multi-aspect assessment of depression-like dynamic performance. The Y-maze test was employed to examine mice's learning and memory capacities. The sociability of mice was assessed using the three-chamber sociability test. The resident intruder paradigm and the light-heat flick were used to evaluate mice’s irritable aggression behavior and pain sensitivity. Lastly, heart rate and blood pressure were measured to examine the activity of sympathetic nerves. Specific procedures are presented in the Supplementary Materials.

### Measurement of *GABAAR* subunits protein levels in *Gabrb2*-knockout mice

Western blot assay was used to measure the expression patterns of *GABAAR Subunits* protein in brain tissues of mice. Radio-immunoprecipitation assay (RIPA) buffer for protein extraction. Brain tissues were comprehensively lysed and homogenized. The protein concentration was determined and modified to appropriate attention for protein expression detection. Subsequent procedures were performed as shown in the Supplementary Materials. The Image Lab 5.2.1 software was employed for analysis.

### Neurotransmitter detection in *Gabrb2*-knockout mice

A HILIC-MS/MS method was adopted to detect neurotransmitter levels in serum as well as the brain tissues of mice according to the procedures described in the Supplementary Materials.

### Electrophysiological recordings

After anesthesia, the abdominal cavities of the animals were opened, and the chest opened upward to expose the heart. Using a syringe, pre-cooled artificial cerebrospinal fluid (ACSF) was injected into the aorta via the left ventricle; the auricular appendix was after that incised for perfusion. After cervical dislocation, the skin was marked, and the skull was opened using a scalpel to expose the whole brain tissues. A medicine spoon was used to harvest the brain tissues and allowed to stand in pre-cooled ACSF (filled with mixed air to saturated condition) for 2 minutes. The brain tissues were placed in a petri dish paved with filter paper. Meanwhile, the ACSF and mixer were transferred onto the petri dish. The tissues containing the cortex were excised using a blade, then glued in the correct direction on the agar-coated plate of the brain-slicing machine. Subsequently, the tissues were immediately transferred to the slicing slot of the brain-slicing machine, fixed, then sectioned into slices of 250 μM thickness. For the cortical regions, specific hippocampal areas of the brain slices were cut off and digested with trypsin (formulated with HBSS) at 33.3° C for 30 min, under high purity oxygen exposure. The trypsin was removed after digestion; after that its effect was terminated by loading each tube with 3 mL oxygenated HBSS Na+ solution. This operation was repeated three times. Subsequently, 2 mL of oxygenated Low Ca2+ HEPES was added, then the tissues were triturated using a Pasteur pipet in the large, medium, and small diameters. After trituration by a Pasteur pipet in large, medium diameters, the tissues were allowed to stand for 2 min. The supernatant was pipetted into a new 15 mL centrifuge tube, followed by 4-5 cycles of trituration with a Pasteur pipet in a small diameter. The mixture was allowed to stand for 2 min then the supernatant was obtained and mixed with the former supernatant harvested above. The mixed cell suspension was seeded into a 3.5 cm polylysine-coated petri dish, 2 mL/dish. The cells were left to stand for 10 min to allow cell adherence to the wall.

### Statistical analysis

All statistical analyses were performed using the IBM SPSS statistical 22 software. Data were expressed in mean ± standard deviation. Comparisons between two groups were performed using one sample unpaired t-test, whereas comparisons between multiple groups were conducted using a one-way analysis of variance (ANOVA). The GABAA receptor current in response to drug treatment and the control GABA assessment (20 μM) were adjusted for data obtained after an electrophysiological examination. Further, we calculated the enhancement drug ratio to the GABAA receptor current. The values of *p < 0.05, **p < 0.01, ***p < 0.001 were considered statistically significant. The GraphPad Prism 8.4 software was used for image plotting.

### Data availability

The data supporting the findings of this study are accessible by the corresponding author upon request.

## RESULTS

### Changes in the emotional state of *Gabrb2* knock-out mice

Assessment of anger-like emotions revealed that the aggressive behavioral scores of *Gabrb2* KO mice were significantly lower than that of WT mice (*p* < 0.01, Figure 1B). The anxiety-like emotions were assessed by open field test (OFT), elevated plus maze (EPM), and light-dark box, which revealed significantly longer total distance and increased mean velocity of *Gabrb2* KO mice compared to the WT mice (*p* < 0.05) and HT mice (*p* < 0.01) and prolonged movement time as compared to the WT mice (*p* < 0.05) and HT mice (*p* < 0.05) in the OFT. Substantial shortened total movement distance was measured in KO mice versus HT mice (*p* < 0.001) and WT mice (*p* < 0.01), whereas notable elevated OT% was found in *Gabrb2* KO mice versus WT mice (*p* < 0.001) and WT mice (*p* < 0.001). In the light-dark box, the entrance times into the bright area were markedly increased in *Gabrb2* KO mice than that in WT mice (*p* < 0.01) but similar to that in HT mice (*p* > 0.05). The depression-like emotions were evaluated by the tail suspension test (TST), FST, and a sucrose preference test; consequently, the immobility time in TST significantly decreased in *Gabrb2* KO mice compared with that in WT mice (*p* < 0.001) and HT mice (*p* < 0.001). The immobility time in the FST of *Gabrb2* KO mice was shortened compared to that of WT mice (*p* < 0.001) and HT mice (*p* < 0.05), whereas that of HT mice was noticeably shorter than that of WT mice (*p* < 0.001). The sucrose preference index of *Gabrb2* KO mice was higher than that of WT mice (*p* < 0.001) and HT mice (*p* < 0.001), (Figure 1C–1H).

### Changes in the somatic state of *Gabrb2* knock-out mice

Further, we evaluated the activity of sympathetic nerves and pain thresholds of mice. Consequently, the KO mice showed a lower heart rate than WT mice (*p* < 0.001), whereas HT mice had a lower heart rate than WT mice (*p* < 0.01). No difference in the diastolic blood pressure was observed among the groups. Nevertheless, the systolic blood pressure of HT mice was remarkably lower than that of WT mice (*p* < 0.01). The tail-flick test demonstrated that the pain threshold of KO mice was notably higher than that of WT mice (*p* < 0.01) and that of HT mice (*p* < 0.001) (Figure 1I, 1J).

### Changes in the social function of *Gabrb2* knock-out mice

Assessment of learning and memory capacities revealed that the KO mice had a reduced spontaneous alternation rate in the Y-maze test than that of WT mice (*p* < 0.05) and HT mice (*p* < 0.05). At the same time, no difference was noted between WT and HT mice (*p* > 0.05). The open arm exploration test revealed that the KO mice (*p* < 0.05) and HT mice (*p* < 0.05) had significantly lower incidences of open arm exploration than WT mice. The sociability of mice was evaluated using the social preference-avoidance test. Unlike WT mice, KO mice revealed a significantly lower preference index for exploring unfamiliar mice (*p* < 0.05). In contrast with the WT mice, *Gabrb2* KO mice (*p* < 0.01) and HT mice (*p* < 0.01) had fewer times exploring new and unfamiliar mice, but not familiar mice. No difference was observed between KO and HT mice (*p* > 0.05). The contact duration of KO mice with unfamiliar mice was shorter than that of WT mice (*p* < 0.05), (Figure 1K, 1L).

### *GABAAR* receptor pathway changes in the *Gabrb2* knock-out mice

**Figure 2:**
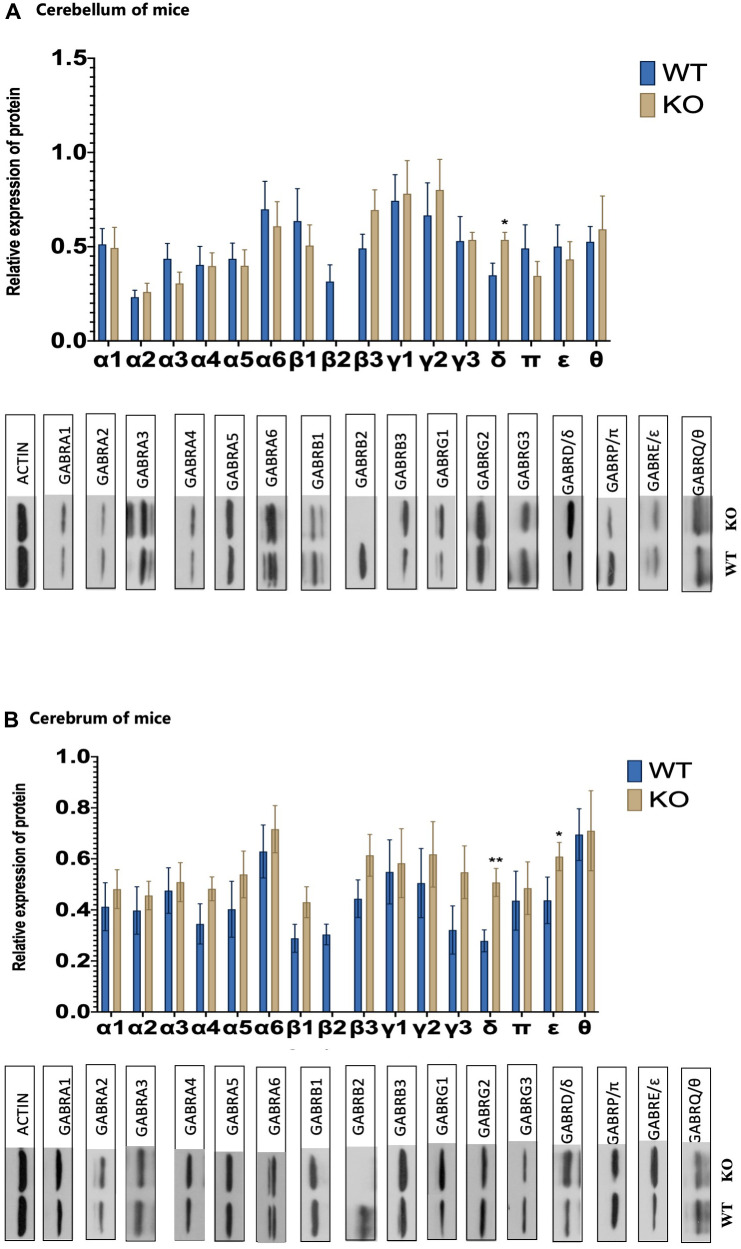
**GABAAR receptor subunits changes.** (**A**) The levels of protein expression for 16 different GABA A receptor subunits of WT and KO mouse in the cerebrum (WT male =8, KO male= 8). (**B**) The levels of protein expression for 16 different GABA A receptor subunits of WT and KO mouse in the cerebellum (WT male =8, KO male=8). Statistical analysis was performed using one-way ANOVA with Newman–Keuls post-hoc test. Average y values ± SEM in the different plots are represented by horizontal bars. *p < 0.05, **p < 0.01, ***p < 0.001.

Western blot assay was used to measure the expression patterns of *GABAA* receptor α1-6 subunits (*GABRA1*, *GABRA2*, *GABRA3*, *GABRA4*, *GABRA5*, and *GABRA6*), *β1-3* subunits (*GABRB1*, *GABRB2*, and *GABRB3*), *γ1-3* subunits (*GABRG1*, *GABRG2*, and *GABRG3*), δ subunit (*GABRD*), ε subunit (*GABRE*), π subunit (GABRP), and θ subunit (*GABRQ*). Protein levels of *GABAA* receptors δ and ε subunits were substantially increased in the cerebral regions. GABAA receptor δ subunit protein expression significantly increased in the cerebellum of *Gabrb2* KO mice compared to WT mice (*p* < 0.05), (Figure 2).

**Figure 3:**
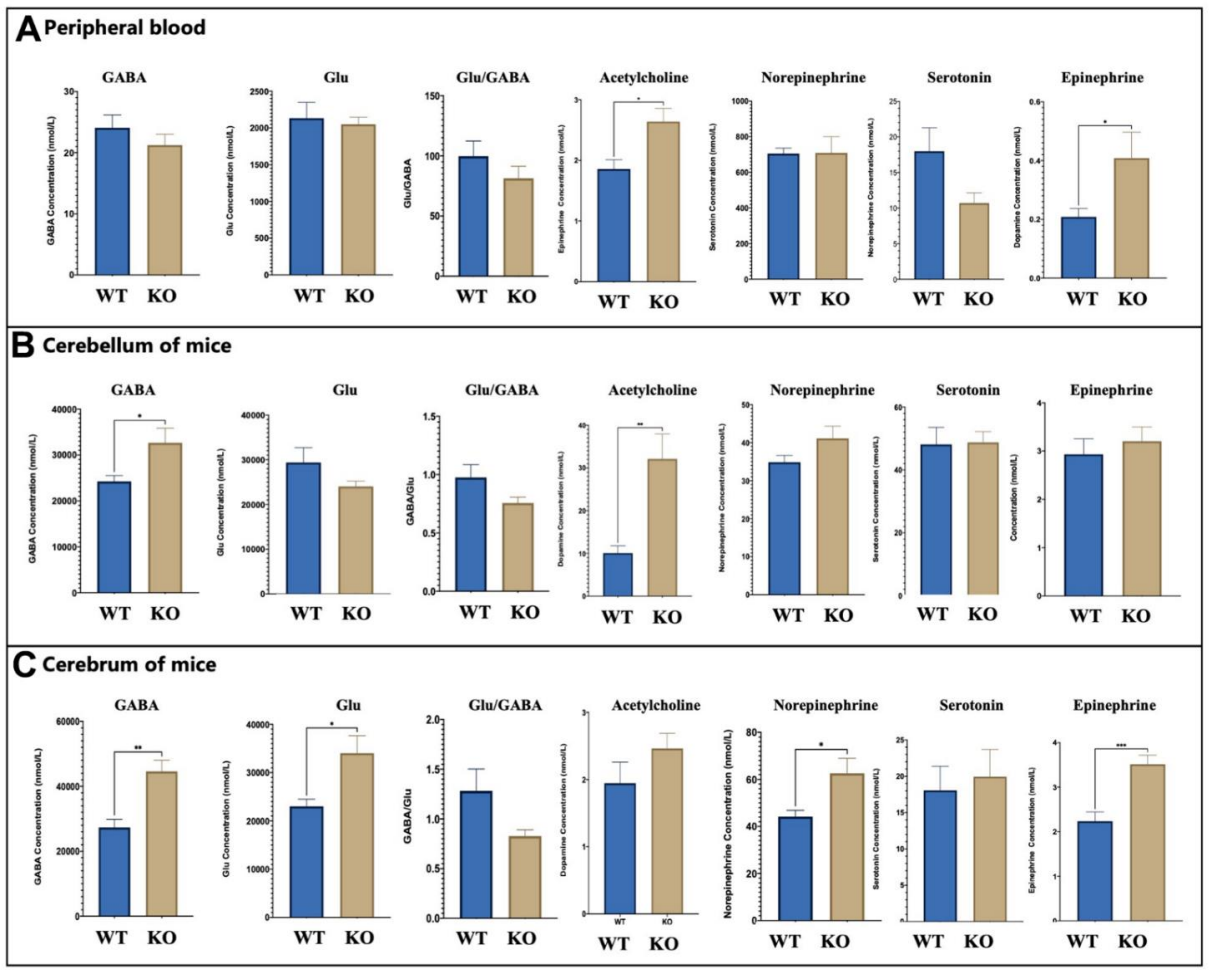
**Neurotransmitters changes.** (**A**) The level of key neurotransmitters *in vivo* of WT and KO mouse (WT male =8, KO male= 8). (**B**) The levels of WT and KO mouse in the cerebrum (WT male =8, KO male= 8). (**C**) The levels of neurotransmitters of WT and KO mouse in the cerebellum (WT male =8, KO male= 8). The levels of GABA, Glu Statistical analysis was performed using one-way ANOVA with Newman–Keuls post-hoc test. Average y values ± SEM in the different plots are represented by horizontal bars. *p < 0.05, **p < 0.01, ***p < 0.001.

Further, we evaluated the levels of 4-aminobutyric acid (GABA), glutamic acid, dopamine, serotonin, norepinephrine, and epinephrine in the peripheral blood of WT and *Gabrb2* KO mice. As a result, the levels of 4-aminobutyric acid (GABA), glutamic acid, serotonin, and norepinephrine in the peripheral blood of KO mice were similar to that in WT mice (*p* > 0.05). Nonetheless, the levels of dopamine and epinephrine in the peripheral blood of *Gabrb2* KO mice were strikingly higher than that of WT mice (*p* < 0.05). The contents of glutamic acid, norepinephrine, serotonin, and epinephrine revealed insignificant differences in the cerebrum tissues of *Gabrb2* KO mice and WT mice (*p* > 0.05). The GABA, GABA/Glu, and dopamine levels were noticeably increased, whereas that of acetylcholine decreased in the cerebral tissues of *Gabrb2* KO mice relative to WT mice (*p* < 0.05). The acetylcholine was not detected in the cerebellum of *Gabrb2* KO mice, and the levels of dopamine and serotonin in the cerebellum of *Gabrb2* KO mice were similar to that of WT mice (*p* > 0.05). The levels of 4-aminobutyric acid (GABA), glutamic acid, norepinephrine, and epinephrine were significantly higher in the *Gabrb2* KO mice than that in the WT mice (*p* < 0.05). These data indicate that *Gabrb2* KO causes compensatory changes in GABA, dopamine, and acetylcholine. Also, GABA/Glu in the cerebral regions may be related to the behavioral phenotype of transgenic mice (Figure 3).

### Dose effect of ALLO on *Gabrb2* KO mice

**Figure 4:**
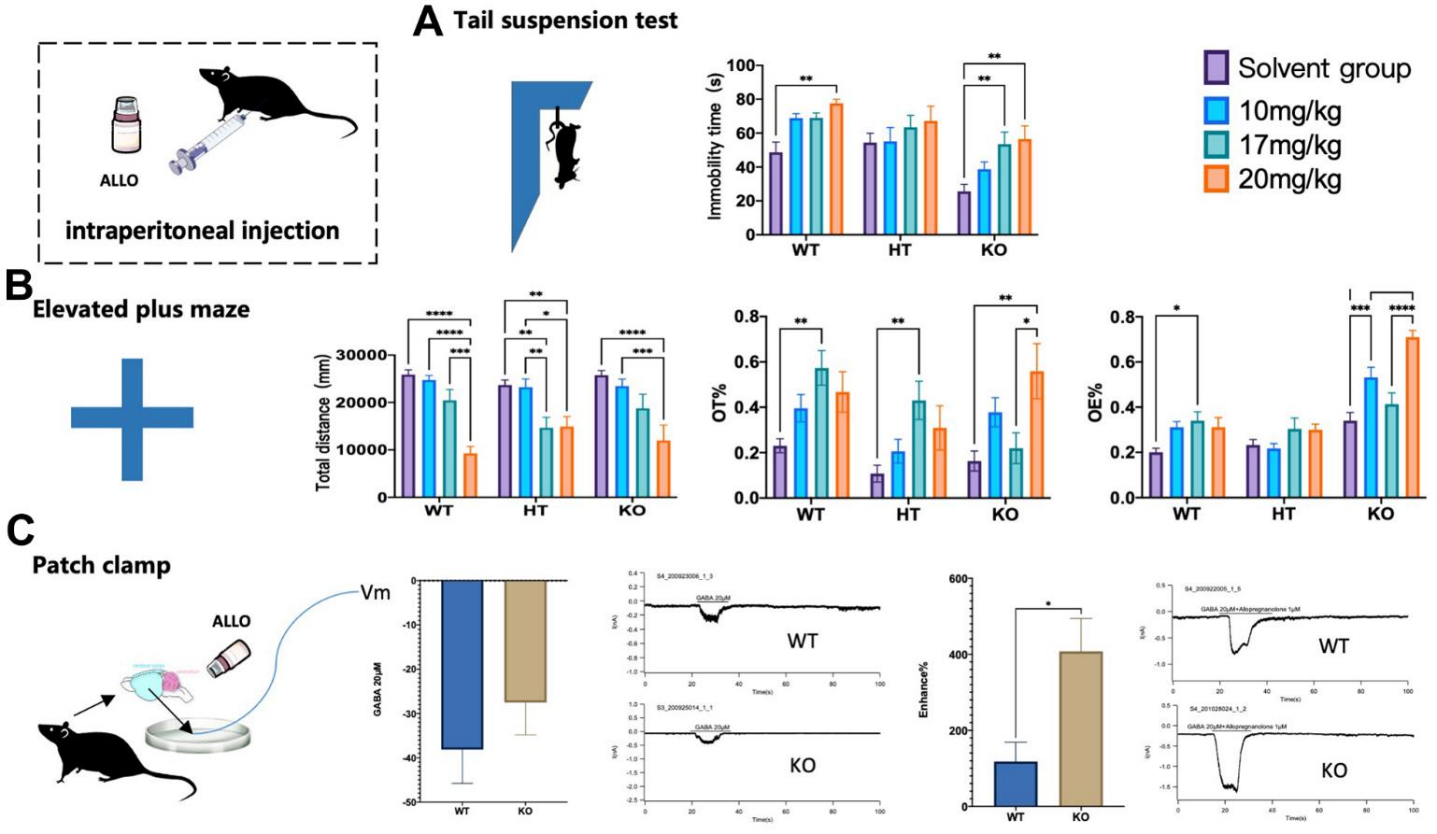
**Changes of KO behavioral phenotypes by ALLO.** The behaviors of WT, HT, or KO mice administered with 10 mg/kg, 17 mg/kg, 20 mg/kg ALLO i.p was compared with that of control mice administered with saline. (**A**) Tail suspension test showing immobility time of mice suspended by the tail to a horizontal bar (WT male: saline=12, 10 mg/kg=12, 17 mg/kg=12, 20 mg/kg=12; HT male: saline= 12,10 mg/kg=11, 17 mg/kg=12, 20 mg/kg=12; KO male: saline=11,10 mg/kg=12, 17 mg/kg=11 20 mg/kg=10). (**B**) Elevated plus maze showing percentile entries and time into open arms (WT male: saline=12, 10 mg/kg=12, 17 mg/kg=12, 20 mg/kg=10; HT male: saline= 12,10 mg/kg=11, 17 mg/kg=12, 20 mg/kg=11; KO male: saline=12,10 mg/kg=12, 17 mg/kg=12, 20 mg/kg=12). (**C**) Patch clamp showing changes of GABAA receptor current in mouse cortical neurons under ALLO intervention between WT male mice and KO male mice (WT male =6, HT male=6, KO male=6). Statistical analysis was performed using one-way ANOVA with Newman–Keuls post-hoc test. Average y values ± SEM in the different plots is represented by horizontal bars. *p < 0.05, **p < 0.01, ***p < 0.001.

The behavioral and cell level changes were observed in mice injected with ALLO. The overall distance of EPM was shortened in a dose-dependent manner after ALLO injection into mice with three genotypes. In response to treatment with 20 mg/kg ALLO, the WT mice significantly covered a shortened total distance. Also, ALLO treatment at doses of 17 mg/kg and 20 mg/kg significantly shortened the total movement distance of HT mice (*p* < 0.01). ALLO treatment at a dose of 20 mg/kg markedly shortened the total movement distance of *Gabrb2* KO mice (*p* < 0.01). Additionally, ALLO treatment at a dose of 17 mg/kg considerably increased the OT% of WT mice and HT mice (*p* < 0.01), and a dose of 20 mg/kg noticeably increased the OT% of KO mice (*p* < 0.01). Furthermore, 17 mg/kg ALLO augmented the OE% of WT mice (*p* < 0.05), whereas 10 mg/kg and 20 mg/kg ALLO significantly augmented the OE% of WT mice (*p* < 0.01). In the TST test, the mice of three genotypes were injected with different doses of ALLO. The immobility time of HT mice was unaffected (*p* > 0.05), whereas that of WT mice was prolonged following treatment with different doses of ALLO, among which 20 mg/kg had a significant effect (*p* < 0.01). ALLO treatment at different doses prolonged the immobility time of *Gabrb2* KO mice; unlike 10 mg/kg dose (*p* > 0.05), 17 mg/kg and 20 mg/kg doses exerted a significant effect (*p* < 0.01), (Figure 4A, 4B).

The patch-clamp technique was used to assess the allosteric regulation of ALLO on *GABAA* receptor currents in the cortical neurons of WT and *Gabrb2* KO mice. No difference in receptor currents was noted in the mouse cerebral cortex in response to treatment with 20 μM GABA. Nevertheless, 1 μM ALLO induced a more significant change in cerebral cortex current in *Gabrb2* KO mice than that in WT mice. ALLO enhanced the agonistic effect of the GABA_A_Rs in cortical neurons in the context of β2 subunit deletion. Therefore, *GABAARβ2* subunit deletion did not affect the binding to ALLO. We could not ascertain whether the ALLO binding site was located in the β2 subunit. However, abnormal expression of the GABAARβ2 subunit certainly caused changes in other subunits, thereby affecting the binding to ALLO. This results in abnormal changes in cerebral receptor currents, manifesting as abnormal behavioral changes in mice (Figure 4C).

## DISCUSSION

According to the published relevant research, the open-field test (OFT) is used to test for behavioral symptoms of PMDD rat models; elevated plus maze (EPM) and light dark box (LDB) tests for anxiety [24]; resident-intruder paradigm for irritability [25]; forced swimming test for depression [26]; saccharin preference test for anhedonia; social preference-avoidance test for social withdrawal [27], heart rate (HR) and blood pressure (BP) [28]. As expected, *Gabrb2* KO mice displayed changes in anxiety-like and depression-like emotions in contrast with PMDD symptoms; changes in the social, learning, and memory abilities similar to PMDD symptoms; changes in pain threshold opposite to PMDD symptoms, hence reducing pain sensitivity. The results above corroborate with the previous studies, which focused on schizophrenia-like phenotypes [22]. Our study concentrated on PMDD-like phenotypes and added new tests for assessing pain sensitivity and heart rate/blood pressure related to PMDD [28–30]. These findings confirm that protein function mediated by *Gabrb2* is closely associated with the pathogenesis of PMDD.

Meanwhile, we adopted molecular biotechnology to analyze the compensatory changes of *Gabrb2* KO mice. Recent research shows that anxiety may result in abnormal expression of *α_4_β_2_δ GABA_A_R* [31]. Among *GABA_A_R* subunits, *GABA_A_R* δ and *GABA_A_R* ε subunit proteins were expressed at higher abundance in the brain region of the *Gabrb2* KO mice. In contrast, *GABAA* receptor δ subunit proteins were significantly expressed in the cerebellar areas. Previous research indicates that corticotropin-releasing hormone (CRH) neurons are modulated by neurosteroid tetrahydro-deoxycorticosterone (THDOC) and act on *GABA_A_R*-containing δ subunits which share close associations with anxiety-like behaviors [32]. The δ-Subunit is necessary for Protein Kinase C-dependent effects of neurosteroids on synaptic GABA _A_ receptor inhibition [33]. Besides, *GABAA(δ)R* may promote fear extinction via a route relying on non-synaptic plasticity [34]. Therefore, GABAA(δ)R has high research potential. Moreover, we found a compensatory increase in dopamine and epinephrine levels in the peripheral blood of transgenic mice; compensatory changes in GABA, dopamine, acetylcholine, and GABA/Glu levels in the brain regions; compensatory changes in GABA, glutamic acid, norepinephrine, epinephrine, and GABA/Glu in the cerebellar area of the mice. Therefore, we speculate that most of the above-stated changes may correlate with the behavioral phenotype of transgenic mice. Besides GABA and Glu indicated above, 5-HT, a monoamine neurotransmitter regulates emotions and cognitive functions in the CNS [35]. NE, DA, etc., are important indicators for assessing depression severity [36]. Also, research has demonstrated a strong correlation between dopamine, 5-HT, norepinephrine, and serotonin in the brain with depression-like emotions [37], which is in agreement with our findings [38].

The neuroactive steroid allopregnanolone (ALLO) is an endogenous positive allosteric modulator of GABA type A receptor (GABAAR), which causes the development of mood disorders, including depression, anxiety, and PMDD [39–41]. Nonetheless, it remains unclear whether ALLO mediates *Gabrb2* as a significant subunit [42]. When ALLO was injected into animal experiments, *Gabrb2* KO did not yield the expected effect on the mouse phenotype. Unlike in the WT mice, ALLO treatment improved the agonistic effect on the GABA_A_ receptor in cortical neurons of KO mice, supporting the hypothesis that the ALLO binding site may not be located on the β2 subunit. Changes in mice caused by abnormal expression of β2 subunit may be related to ALLO sensitivity. However, there is an urgent need for additional comprehensive studies.

## CONCLUSIONS

*GABAAR β 2* has a regulatory effect on PMDD-like behaviors with disturbance of neurotransmitters, hence directly or indirectly affecting its mediated ALLO binding in vital brain areas. This causes abnormal nerve synaptic currents, and manifestation of PMDD-related clinical symptoms, which should be validated in future research.
